# Cystic Adventitial Disease of the Common Femoral Artery: A Rare-Case Report

**DOI:** 10.3389/fsurg.2021.814036

**Published:** 2022-01-11

**Authors:** Qilong Wang, Zhihua Cheng, Liang Tang, Qi Wang, Ping Zhang, Hua Zhang

**Affiliations:** ^1^Department of Vascular Surgery, The First Bethune Hospital of Jilin University, Changchun, China; ^2^Department of Neurology, Songyuan Jilin Oilfield Hospital, Songyuan, China; ^3^Department of Hepatopancreatobiliary Surgery, The First Bethune Hospital of Jilin University, Changchun, China

**Keywords:** cystic adventitial disease, common femoral artery, space-occupying lesion, surgical excision, autologous saphenous vein, case report

## Abstract

Herein, we report the case of a 59-year-old man with intermittent claudication of ~100 m, who complained of resting pain in his lower right extremity. A pelvic, contrast-enhanced, computed tomography scan showed the presence of cystic density in the lower segment of the right common femoral artery. Faced with the risk of acute limb ischemia, we navigated a challenging diagnostic procedure to choose an appropriate treatment for him. Additionally, we performed a pathological investigation of the excised common femoral artery following the excision bypass. On postoperative day 5, the patient was discharged from the hospital. During the 2-year follow-up, no new cysts were discovered, and the patient had favorable prognosis.

## Introduction

Cystic adventitial disease (CAD) of the common femoral artery is a rare vascular disorder (1) that is potentially limb-threatening. This condition mainly affects middle-aged men, but it can develop at any age. Until recently, the most common blood vessel affected by CAD was the popliteal artery (2), while the common femoral artery was rarely involved. An involved common femoral artery might produce an arterial embolism in a short period of time, resulting in severe limb ischemia and risking limb loss. We present a case that we believe is unique, as the patient's common femoral artery was nearly completely blocked. In this patient, arterial thrombosis rapidly produced acute limb ischemia with no distinguishing clinical signs equivalent to arterial embolism. However, treatment approaches for CAD of the common femoral artery vs. arterial thrombosis are contrary. The specific CAD presentation is easily misdiagnosed and overlooked. Accurate diagnosis frequently determines the prognosis; thus, careful attention should be paid to properly diagnosing CAD of the common femoral artery. Self-cure is usually not an option. Surgical excision is currently the main treatment method, which can be carried out via vascular resection and revascularization. Histopathological examination of tissue samples is the gold standard to determine the final, accurate diagnosis. The patient discussed in this case report had an excellent prognosis following vascular excision and revascularization with an autologous saphenous vein, and showed good results after a 2-year follow-up. In this paper, we present a unique instance of CAD of the common femoral artery, details on the therapeutic options for this patient, and an analysis of surgical treatment results.

## Case Presentation

A 59-year-old man, with intermittent claudication of ~100 m, complained of resting pain in his lower right extremity. As his symptoms deteriorated, he was immediately transferred to our institution for further investigations for an accurate diagnosis and surgical treatment. His clinical symptoms included cold right limbs, most severely at the toes; pale skin; numbness; paresthesia; and intermittent claudication. There was no pulse in the right common femoral artery, the right dorsal artery of the foot, and the right posterior tibial artery. The pulsation of each blood vessel in the left lower extremity was palpable. His medical history was notable for drinking and smoking. The patient denied any history of other medical conditions, operations, and hereditary disease.

**Figure 1:**
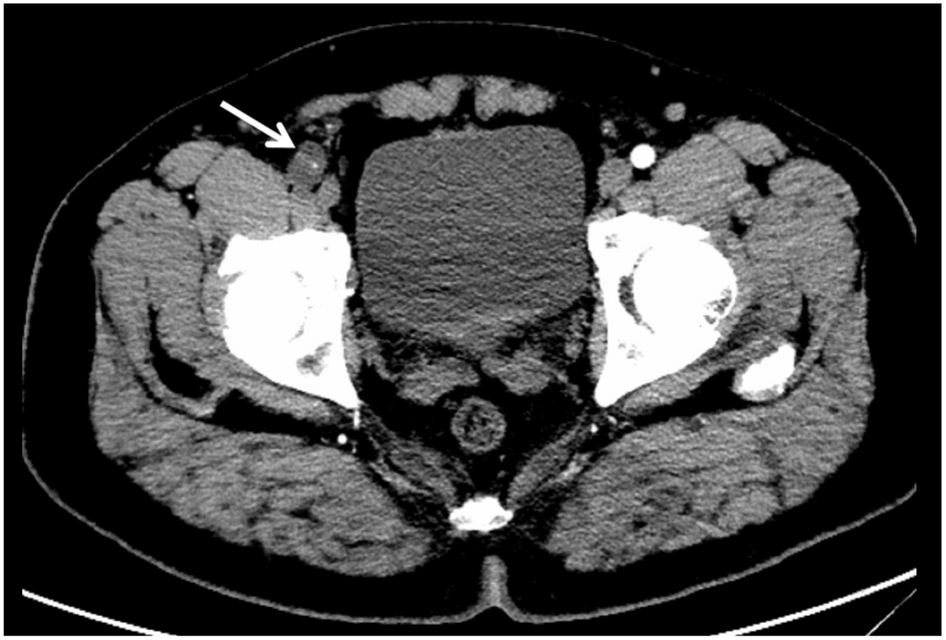
Preoperative, pelvic, contrast-enhanced CT imaging showed a low-density shadow next to the common femoral artery, and the common femoral artery was almost completely occluded (white arrow).

Upon admission, the patient had a temperature of 36.2°C, heart rate of 78 beats/min, blood pressure of 125/75 mmHg, and respiratory rate of 16 breaths/min. Routine examination and tumor-markers were within the normal ranges. Ultrasonography revealed a cystic mass in the lower segment of the right common femoral artery. The local lumen was severely stenosed, and the blood flow rate was slowed. Furthermore, pelvic, contrast-enhanced computed tomography (CT) showed a cystic density, which was ~3 × 2 cm^2^ in size. The common femoral artery was almost completely occluded (Figure 1). It was identified as a common femoral-arterial, space-occupying lesion based on the preoperative medical history, physical findings, and auxiliary examination. However, following excision, a pathological investigation was required to determine the properties of the lesion.

**Figure 2:**
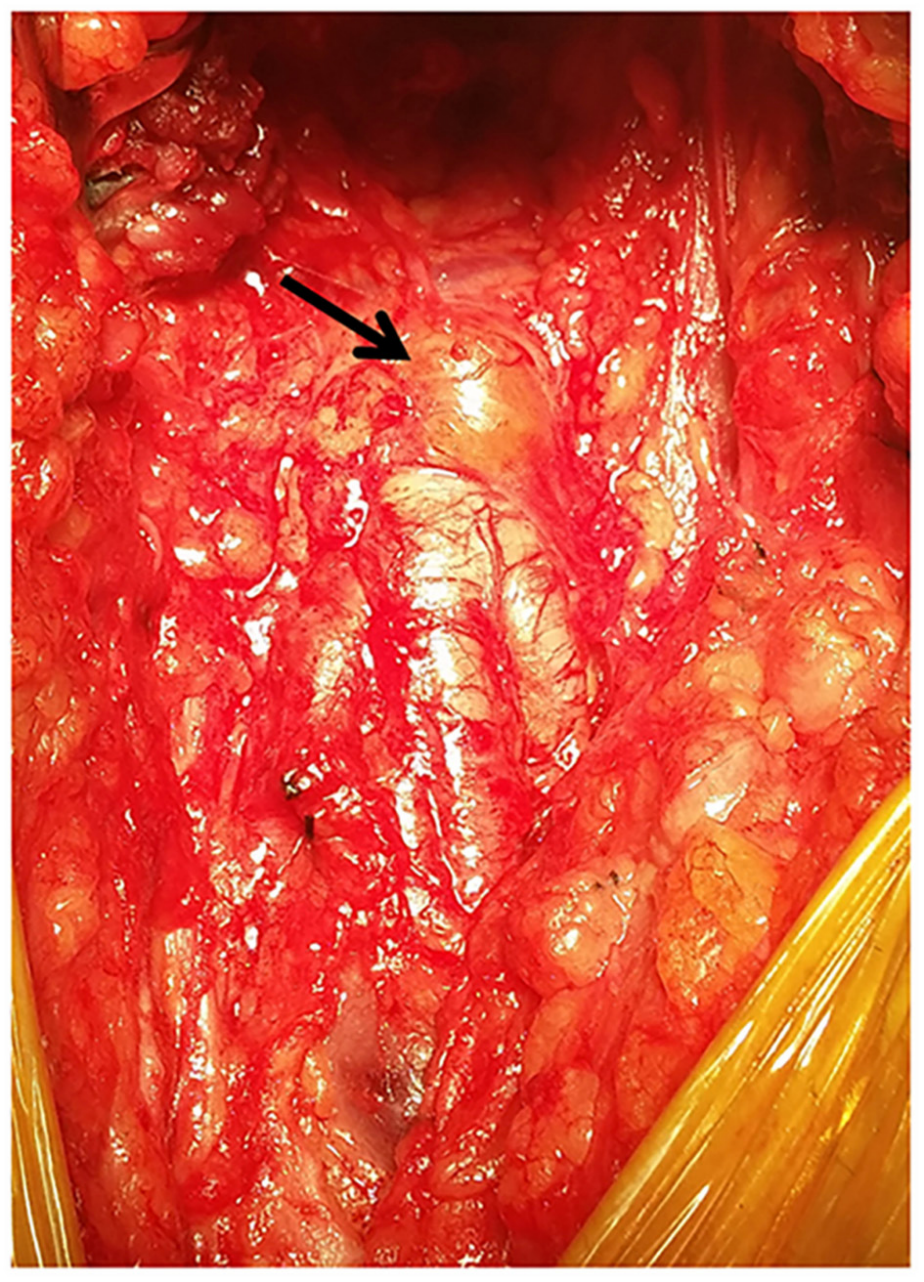
Intraoperative photograph showing the adventitial cyst on the anterior wall of the common femoral artery (black arrow).

**Figure 3:**
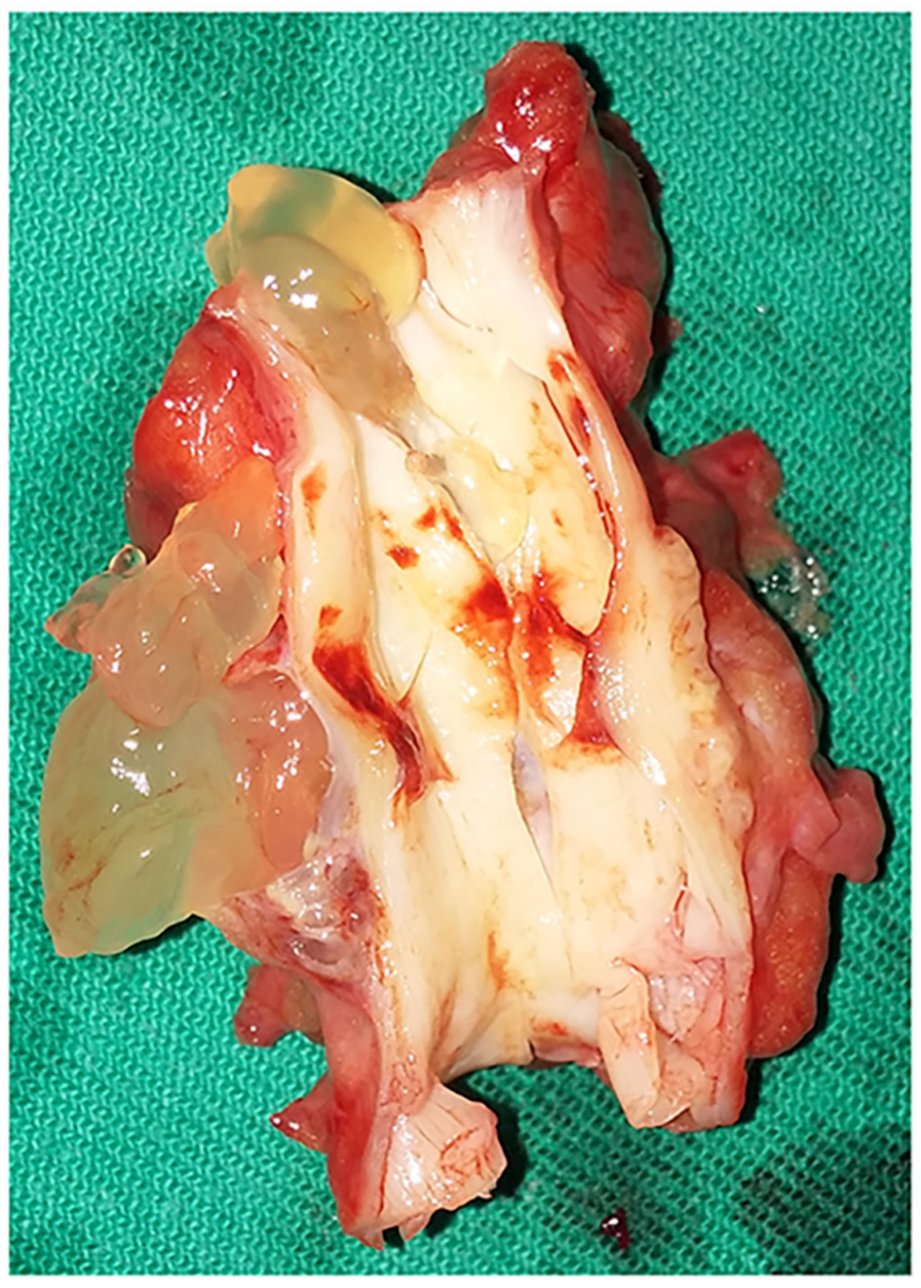
Adventitial-cyst specimen.

In view of the above diagnosis, a surgical resection of the space-occupying lesion was performed. A 10-cm incision was made along the right groin, and the subcutaneous tissue and muscle were gradually separated. The right common femoral artery was gradually exposed, and the aberrant common femoral artery anatomy was revealed. Intraoperatively, the femoral artery was bulging irregularly in the form of a vesicle, and the adventitia was translucent (Figure 2). After excision, it was found that the arterial intima was intact, and the vessel wall was locally thickened. A pectin-like material filled the space between the layers of vessel adventitia, causing luminal stenosis (Figure 3).

Following surgical excision of the diseased blood vessel, we decided to perform autologous saphenous vein reconstruction. The blood flow of the autologous saphenous vein was normal postoperatively, and the distal artery exhibited excellent pulsation. Following surgery, the patient recovered quickly. On the 5th postoperative day, the patient was discharged from the hospital. His prognosis was favorable. The patient remained asymptomatic during the postoperative 1-month, 6-month, 1-year, and 2-year follow-ups. The patient was urged to return for a 6-month check-up.

## Discussion

**Figure 4:**
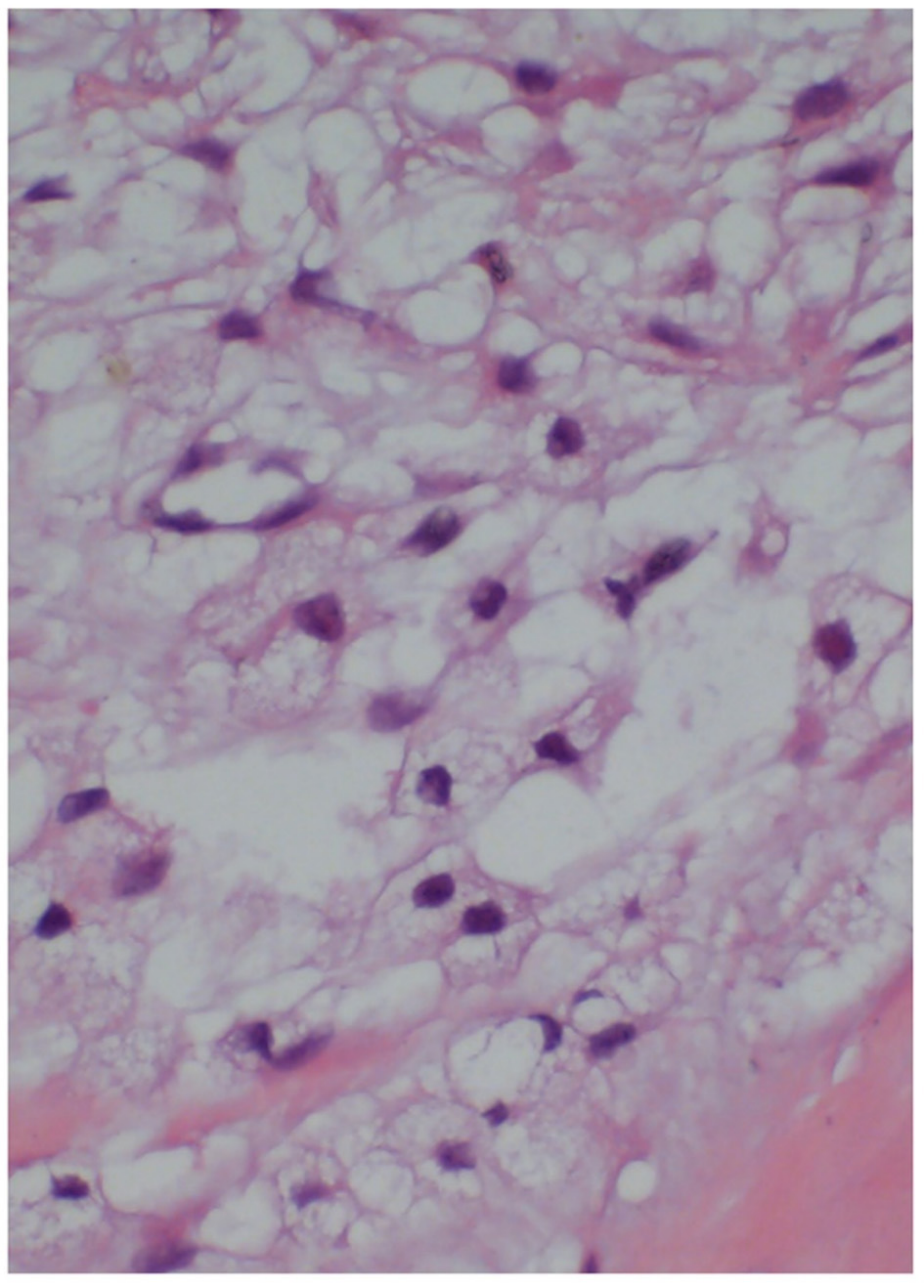
Histopathological examination of the common femoral artery (stained with hematoxylin and eosin) showed mucus degeneration, mucus retention, and cystic changes in the common femoral artery wall.

CAD is an uncommon (3–5), non-atherosclerotic cause of peripheral artery disease that is distinguished by the production of mucinous cysts in the adventitial layer of arteries. Alkins and Key published the first case of CAD in 1947 (3). The majority of published literature thus far have reported popliteal-artery CAD as the cause for systemic CAD (2, 6), while common femoral-artery CAD is quite rare (7, 8). Until now, only a few cases of CAD have been reported to occur in the common femoral artery. In the early stages, there are no obvious clinical indications in the limb. As a result, it is usually overlooked. Intermittent claudication is a common, initial clinical sign. Cold limbs, pale skin, numbness, paresthesia, rest pain, calf muscle atrophy, lack of peripheral artery pulse, and even gangrenous ulcers are frequent, late-stage symptoms of limb ischemia. Because of the rarity of CAD of the common femoral artery, its etiology, pathophysiology, clinical symptoms, and imaging findings have not been clarified. Therefore, it is difficult to distinguish the CAD of the common femoral artery from other common femoral-arterial diseases such as arteriosclerotic obliterans, arterial embolism, polyarteritis, thromboangiitis obliterans, nodular arteritis, and arteriosclerotic lesions (e.g., aneurysms). Duplex ultrasonography can provide an accurate basis for these differential diagnoses. Given its non-invasive and portable characteristics, duplex ultrasonography is usually the first choice for the examination of adventitia cysts. It can clearly indicate the femoral artery adventitia cyst's location, size, form, and compression. The mass is measurable and slender. There is no blood flow signal in the mass. When the femoral artery is compressed, it shows that the femoral artery lumen becomes narrow and the blood flow velocity increases. This patient's clinical symptoms were consistent with those of an acute arterial embolism. In fact, we misdiagnosed his disease once. The potential to misdiagnose CAD of the common femoral artery merits our consideration and contemplation. We believe that there are different supplemental examinations available to accurately diagnose CAD, aside from clinical symptoms. Early detection and treatment of this illness can help prevent worsening thrombosis and ischemia. Some of the tests available to diagnose CAD are ultrasonography (9, 10), magnetic resonance angiography (11, 12), and computed tomography angiography. However, histopathological analysis of the excised material is the gold standard for a definitive diagnosis. In this patient, mucus degeneration, mucus retention, and cystic changes in the common femoral artery wall were observed. The morphology revealed evidence of vascular cystic adventitia degeneration (Figure 4).

There have been four theoretical opinions about the etiology of CAD over the years, but they are all based on clinical-pathological investigations. It is difficult to coherently explain the occurrence of cysts in diverse sites. According to the injury hypothesis, chronic and degenerative illness is produced by repetitive trauma (9). Per the ganglion-cyst hypothesis, larger synovial cysts can often be seen spreading along blood vessel branches, finally invading the blood vessel adventitia (13). According to the system-disorder hypothesis, there is a manifestation of systemic connective tissue diseases (14). The development hypothesis states that during embryonic development, mucus-secreting mesenchymal cells from adjacent joints penetrate the adventitia of the artery to release mucus in the future (15, 16). Although each hypothesis has supporting evidence, no single explanation can account for all situations. Therefore, further exploration is needed.

Presently, there is no generally acknowledged therapy for CAD of the common femoral artery due to the limited number of patients with this disease. The non-surgical approach is to puncture the cystic cavity using ultrasound- or computed tomography-guided percutaneous aspiration (17). Although aspiration can remove a large amount of cystic fluid and restore blood flow to normal rates, it can easily recur because the mesenchymal cells that secrete mucin still exist. As a result, this approach is generally inapplicable. This sort of surgery is only appropriate for patients who refuse surgery or are unable to endure it. Surgical excision including cystectomy, vascular resection, and revascularization is currently the main treatment strategy (17, 18).

The term “CAD cystectomy” was coined by Holmes (19). In this surgical method, the diseased vessel is exposed, the outer membrane is cut longitudinally, cystic fluid is removed, and the cyst wall is peeled-off. Intraoperative cystectomy was attempted on this patient, but there was no improvement in the patency of the vascular stenosis. For this patient, vascular excision and revascularization with an autologous saphenous vein was performed. We believe that this should now be the primary technique to ensure vessel patency. Because of the recurrence of cysts created by mucin-secreting cells following surgery, artery-bypass surgery may result in a decreased need for re-intervention. During the 2-year follow-up, no new cysts were discovered in this patient. The patient's prognosis following the procedure was very good.

We hypothesize that accurate removal of the damaged tissue may be the key to long-term healing that does not exacerbate the illness. Because of the small number of CAD cases and the short follow-up time in this case, the next step to further explore the origin and pathophysiology of this disease—and to discover treatments with less trauma and a low recurrence rate—should be a multi-center, prospective, clinical study. The exact surgical approach for an individual patient should be chosen based on their condition. Regular follow-up examinations following the operation are also essential.

## Conclusion

CAD of the common femoral artery is an uncommon condition, and it is hence difficult to distinguish CAD of the common femoral artery from other diseases. The primary treatment option is currently surgical excision, which can be accomplished by vascular resection and revascularization. The gold standard for the ultimate, definitive diagnosis of CAD is histopathological inspection of the removed material. In the future, we should consider the possibility of CAD of the common femoral artery in the differential diagnosis of any common femoral arterial disease.

## Data Availability Statement

The original contributions presented in the study are included in the article/supplementary material, further inquiries can be directed to the corresponding author/s.

## Ethics Statement

The studies involving human participants were reviewed and approved by the First Bethune Hospital of Jilin University. The patients/participants provided their written informed consent to participate in this study. Written informed consent was obtained from the individual(s) for the publication of any potentially identifiable images or data included in this article.

## Author Contributions

QilW evaluated the patient, initiated the case report, reviewed the literature, and drafted the manuscript. HZ, ZC, LT, and PZ consulted the relevant literature and participated in the diagnosis and treatment of the patient. QiW, HZ, and PZ were responsible for formulating the patient's treatment plan and revising the manuscript. All authors issued final approval of the submitted manuscript.

## Funding

This study was financially supported by the Natural Science Foundation of Jilin Province, China (Nos. 20200201353JC, 20210204157YY, and 20210101276JC).

## Conflict of Interest

The authors declare that the research was conducted in the absence of any commercial or financial relationships that could be construed as a potential conflict of interest.

## Publisher's Note

All claims expressed in this article are solely those of the authors and do not necessarily represent those of their affiliated organizations, or those of the publisher, the editors and the reviewers. Any product that may be evaluated in this article, or claim that may be made by its manufacturer, is not guaranteed or endorsed by the publisher.
